# Dermoscopy of Mammary Paget’s Disease: A Case Report and Literature Review

**DOI:** 10.7759/cureus.91645

**Published:** 2025-09-05

**Authors:** Niharika Singh, Vishal Gaurav, Sonika Garg, Varniraj Patel, Nita Khurana

**Affiliations:** 1 Dermatology and Venereology, Maulana Azad Medical College, New Delhi, IND; 2 Dermatology, Maulana Azad Medical College, New Delhi, IND; 3 Dermatology and Venereology, All India Institute of Medical Sciences, Rajkot, Rajkot, IND; 4 Pathology, Maulana Azad Medical College, New Delhi, IND

**Keywords:** bowen’s disease, dermoscopy, immunohistochemistry, mammary paget’s disease, nipple carcinoma

## Abstract

Mammary Paget’s disease (MPD) is a rare intraepidermal adenocarcinoma affecting the nipple-areolar complex, often misdiagnosed as benign dermatoses like eczema or psoriasis. We report a case of a 63-year-old postmenopausal woman presenting with a one-month history of an erythematous, eczematous plaque over the right nipple-areolar region. Clinical examination revealed partial destruction of the nipple without an underlying breast mass. Dermoscopic evaluation showed pink structureless areas, white scales, and shiny white lines (chrysalis-like structures), suggestive of MPD. Histopathology demonstrated pale-staining Paget cells within the epidermis, and immunohistochemistry confirmed the diagnosis with strong positivity for cytokeratin 7 and human epidermal growth factor receptor 2 (HER2), and negativity for CK5/6, ruling out Bowen’s disease. Imaging (mammography, ultrasound, and MRI) showed no evidence of underlying carcinoma. In light of the patient's refusal of surgical intervention, she was treated with topical imiquimod 5% cream thrice weekly for 16 weeks, with moderate clinical improvement. A comprehensive literature review of 16 reported cases revealed variable dermoscopic features depending on pigmentation, with non-pigmented cases showing pink/white structureless areas, white scales, and shiny streaks-features evident in our case. Differentiation from mimics like eczema, psoriasis, and Bowen’s disease using dermoscopy is essential for early diagnosis. This case emphasizes the utility of dermoscopy in identifying MPD, even in non-pigmented cases without associated carcinoma. It also highlights the potential role of non-invasive therapies like topical imiquimod in managing selected patients. A clinico-dermoscopic-pathologic correlation is crucial to avoid misdiagnosis and ensure timely treatment. Regular follow-up is recommended to monitor therapeutic response and detect any future neoplastic transformation.

## Introduction

Mammary Paget’s disease (MPD) is a rare form of breast cancer, an intraepidermal adenocarcinoma, that primarily involves the nipple-areolar complex (NAC) and is frequently associated with underlying ductal carcinoma in situ or invasive ductal carcinoma [1]. Clinically, MPD often mimics benign dermatoses such as eczema or psoriasis, leading to diagnostic delays. While most cases occur in the setting of an underlying malignancy, a minority present without any detectable carcinoma, posing a diagnostic challenge [2]. Dermoscopic evaluation, though underutilized, can provide important non-invasive diagnostic clues by revealing vascular and structural patterns that differentiate MPD from other dermatoses [3-5]. However, literature on dermoscopic features of MPD, particularly in non-pigmented cases or those lacking underlying carcinoma, remains limited.

This report describes a case of MPD in a postmenopausal woman without associated breast malignancy, highlighting the utility of dermoscopy, histopathology, and immunohistochemistry in early diagnosis. Furthermore, it explores the potential role of topical imiquimod as a non-surgical treatment option.

## Case presentation

**Figure 1:**
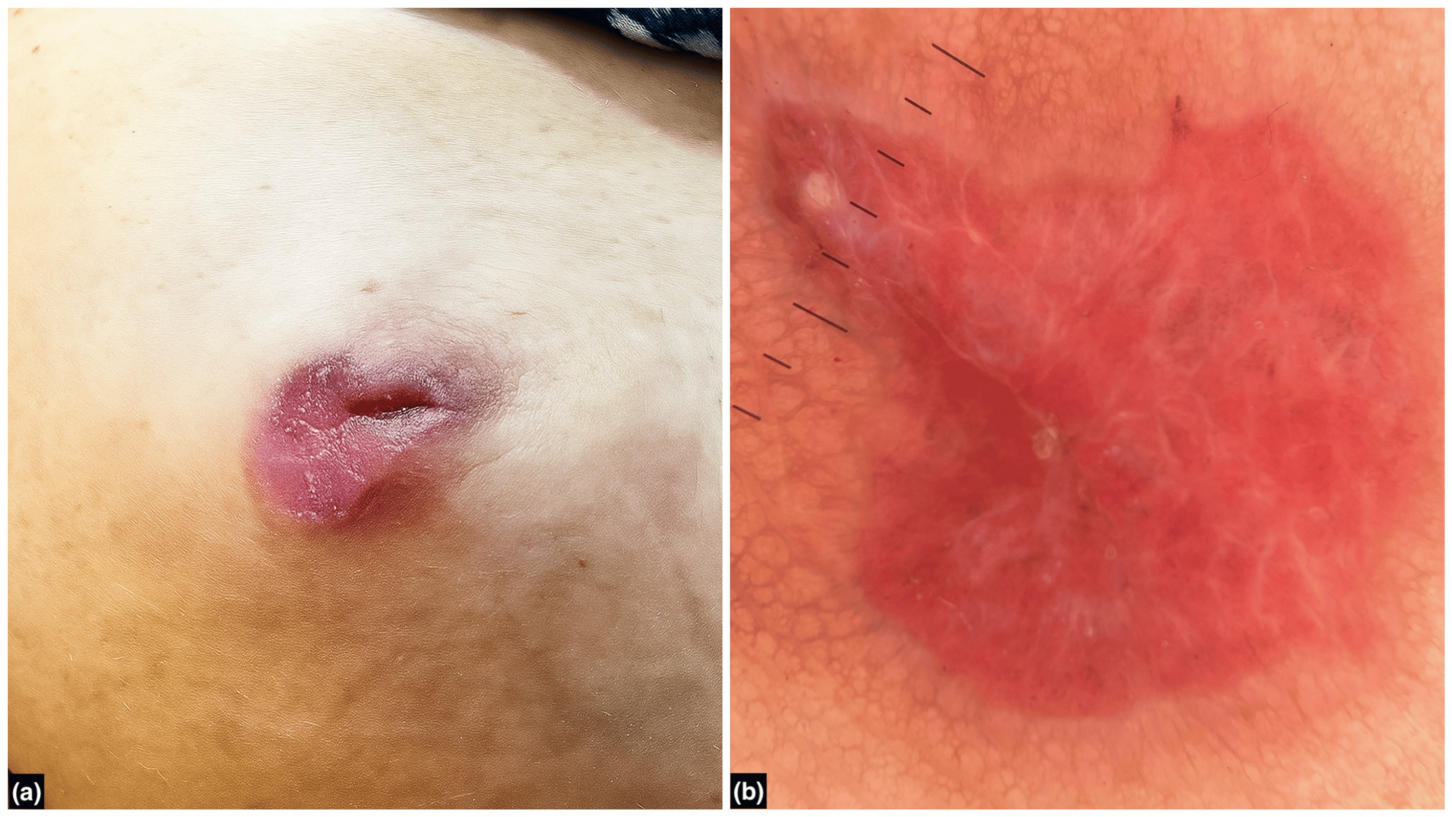
Clinical and dermoscopic features of the erythematous plaque on the right nipple. (a) A 3 cm x 3 cm single, well-to-ill-defined, irregularly-shaped erythematous plaque on the right nipple with mild scaling; (b) Dermoscopy (Heine DeltaONE, HEINE Optotechnik GmbH & Co. KG, Gilching, Germany) using polarized light at 10x magnification revealed pink structureless areas, white scales, and white shiny lines (chrysalis-like structures).

A 63-year-old postmenopausal, nulliparous woman presented with a one-month history of an asymptomatic plaque over the NAC of the right breast (Figure 1a). The lesion began as a small area of erythema, which progressed over one week to develop fine scaling. Over the subsequent three weeks, it enlarged to its current size and developed a more defined, eczematous texture.

There were no complaints of pain, itching, burning, bleeding, or discharge. Her medical history included well-controlled type 2 diabetes mellitus for the past five years; there was no personal or family history of malignancy or dermatological disorders. She had not used any topical or systemic therapies (including corticosteroids or emollients) prior to presentation.

On examination, a 3 cm × 3 cm erythematous plaque with indistinct margins was noted over the right nipple, associated with partial destruction of the NAC and nipple inversion. There was no evidence of slough, tenderness, discharge, or underlying breast mass. Axillary lymph nodes were not palpable. Based on clinical features, a provisional diagnosis of MPD was considered, with differentials including Bowen’s disease, eczema, and psoriasis.

**Figure 2:**
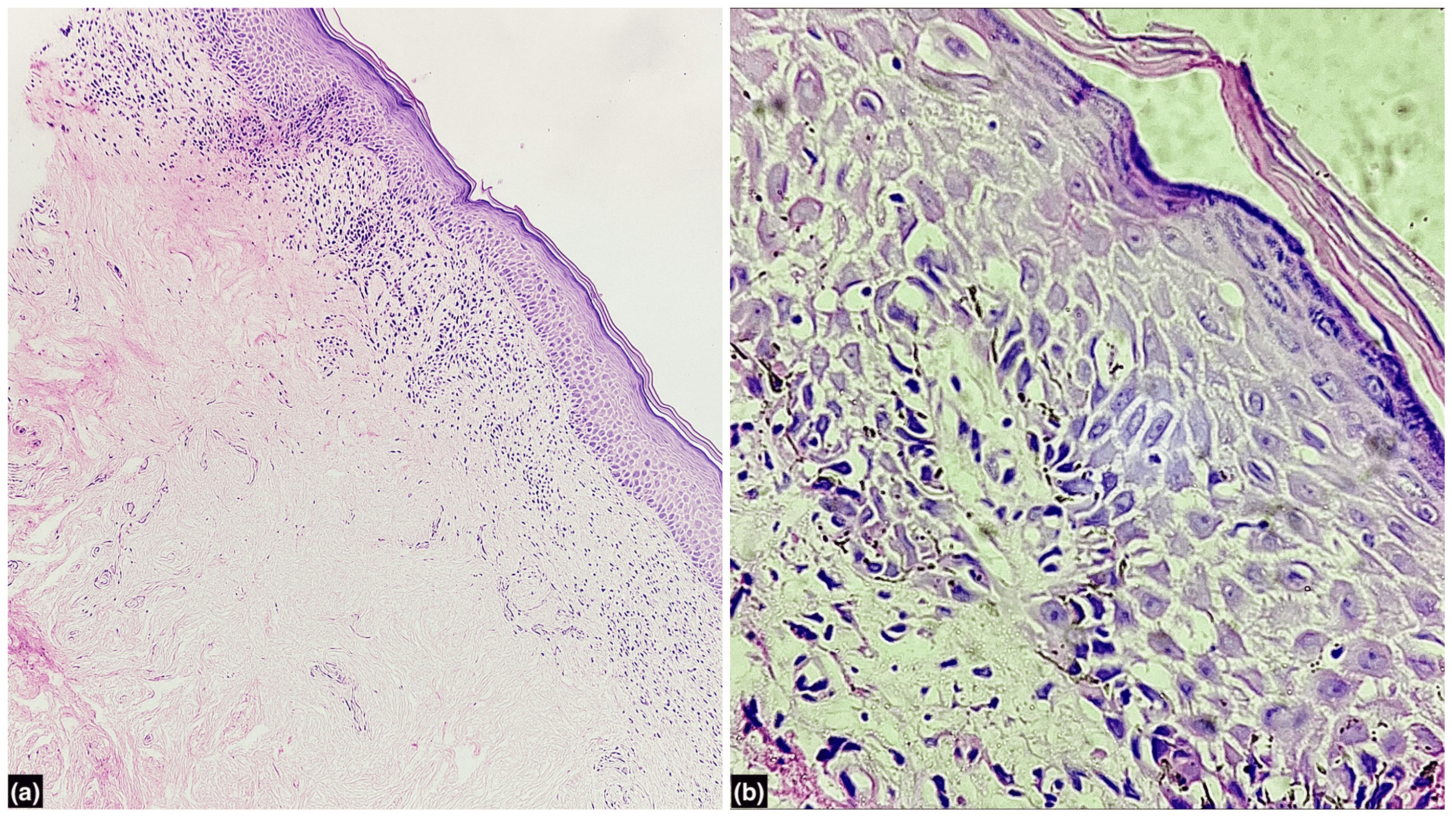
Histopathological examination of a skin biopsy from the lesion Histological section showing (a) mild to moderate lymphocytic infiltrate in the upper dermis with pigment incontinence (Hematoxylin and eosin, 100x); (b) basal layer and malpighian layer showing scattered cells with large nuclei and pale cytoplasm (Hematoxylin and eosin, 400x).

Dermoscopy revealed pink structureless areas, white scales, and white shiny lines (chrysalis-like structures) (Figure 1b), supporting the clinical suspicion. Histopathological examination of a skin biopsy from the lesion showed mild to moderate lymphocytic infiltrate in the upper dermis with pigment incontinence (Figure 2a) along with pale-staining cells with hyperchromatic nuclei irregularly distributed within the epidermis (Figure 2b).

**Figure 3:**
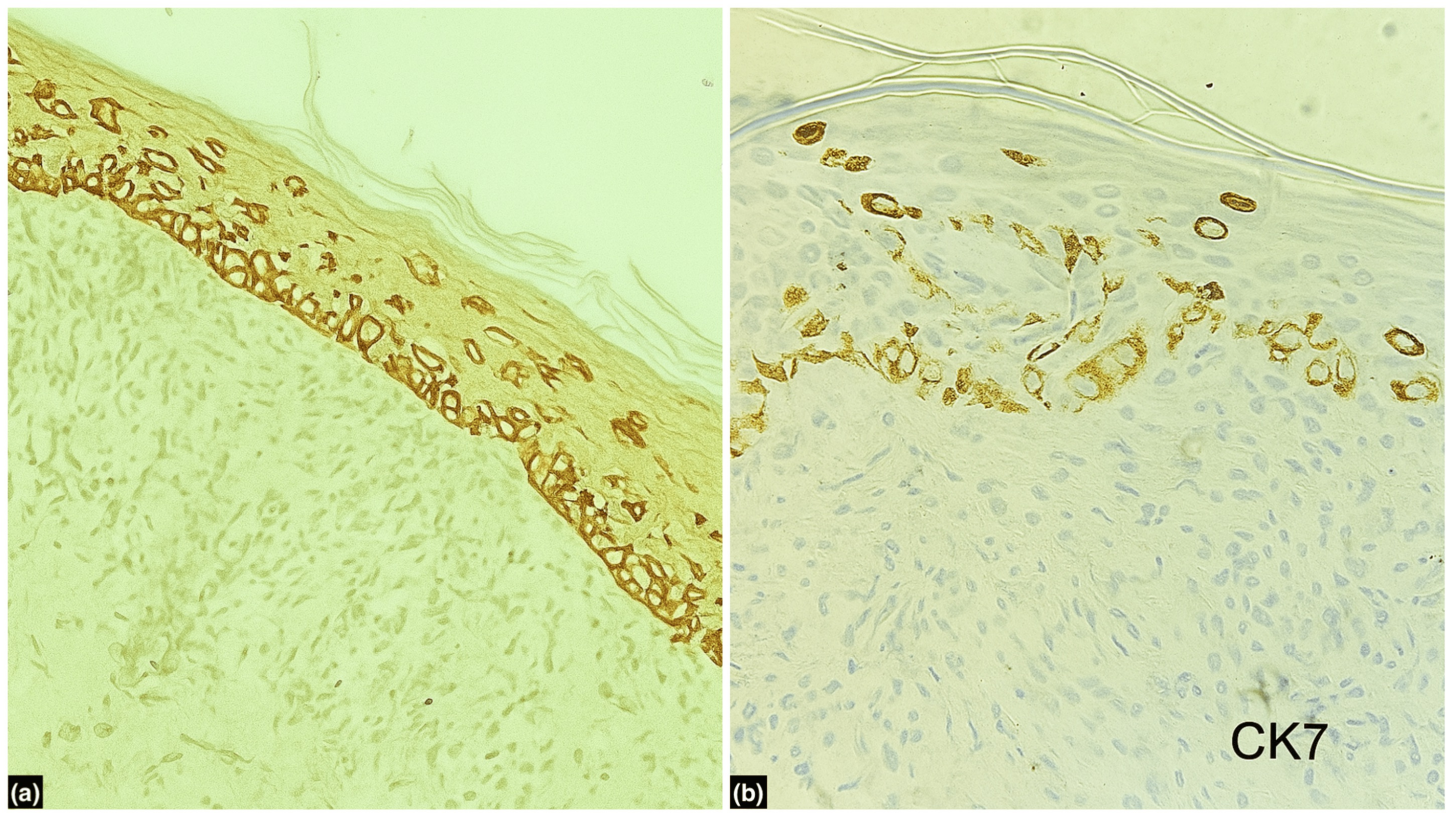
Immunohistochemistry showing (a) HER2-positive cells in the epidermis (100x) and (b) CK7-positive intraepidermal cells (400x). HER2: human epidermal growth factor receptor 2; CK7: cytokeratin 7

Immunohistochemistry demonstrated strong positivity for HER2 (Figure 3a) and cytokeratin 7 (Figure 3b), and negativity for cytokeratin 5/6, thereby confirming the diagnosis of MPD and excluding Bowen’s disease.

Mammography, breast ultrasound, and contrast-enhanced MRI were performed to assess for any underlying malignancy. All imaging studies were normal, confirming the absence of an associated carcinoma. After thorough counseling, the patient declined surgical intervention. Consequently, she was prescribed topical imiquimod 5% cream to be applied three times per week for 16 weeks as a non-invasive alternative. At the end of 16 weeks, a follow-up consultation was conducted via telemedicine. The patient reported an estimated 60-70% reduction in size, erythema, and scaling. The patient was educated about regular breast self-examinations and scheduled for periodic follow-up to monitor treatment response and screen for any future breast malignancy.

## Discussion

The pathogenesis of MPD is incompletely understood at present. Two prevailing theories have been proposed to explain its pathogenesis. The epidermotropic theory suggests that malignant ductal cells migrate to the epidermis via lactiferous ducts. In contrast, the transformation theory proposes that Paget cells arise from keratinocytes within the epidermis that undergo malignant transformation independently of any underlying neoplasm [1,2]. The latter hypothesis explains the small subset of MPD cases, including the present case, in which no underlying malignancy is detected.

Clinically, MPD may mimic benign inflammatory dermatoses such as eczema or psoriasis, often leading to misdiagnosis and delayed treatment [1]. Dermoscopy is a valuable, non-invasive diagnostic tool in such situations, as it reveals morphological patterns that may not be apparent on clinical examination. In pigmented MPD, dermoscopy shows brown peppering, gray-blue dots, irregular pigment networks, and polymorphous vessels - features that may simulate melanoma. Additionally, regression-like structures and blue-white veils have been reported in some pigmented variants [3-9]. In contrast, non-pigmented MPD typically shows pink structureless areas, white scales, white shiny streaks (also known as chrysalis-like structures), and irregular dotted or linear vessels, which reflect chronic epidermal and vascular changes [10-13].

A structured literature search was performed using PubMed and Google Scholar databases to identify published cases of mammary Paget’s disease featuring dermoscopic evaluation. The search terms included combinations of “mammary Paget’s disease,” “dermoscopy,” “dermatoscopy,” and “nipple areolar complex.” Articles published up to June 2025 were considered. Case reports, case series, and original studies that provided clinical and dermoscopic descriptions of MPD were screened. A total of 16 relevant articles with clear documentation of dermoscopic findings were included in the final analysis. These reports have been summarized in Table 1, which outlines patient demographics, clinical appearance, and dermoscopic patterns [3-18].

**Table 1:** Articles on dermoscopic features of mammary Paget’s disease (MPD) NAC: nipple-areolar complex

Author, Year	Age of Patient(s)	Clinical Presentation	Dermoscopy Findings
Hida T et al., 2012 [3]	36, 74, 81 years	Irregular black-brown macules, one with nipple retraction	Reticular pigmentation, irregular black dots, regression structures mimicking melanoma
Longo C et al., 2007 [4]	70 years	Pigmented plaque (5.5 × 4 cm) with thin, scaly surface on the left breast	Diffuse light brown pigmentation, irregular black dots, small gray-blue structures, irregular vessels
Yanagishita T et al., 2011 [5]	37 years	Hyperpigmented, non-painful macule (4 × 4 mm) with irregular borders	Irregular blue-white structures, irregular dots/globules, peppering-like blue-gray dots, dotted and corkscrew vessels
Brugués A et al., 2015 [6]	73 years	Pigmented ill-defined patch (8 × 3 mm) on left nipple	Central: Atypical black network, gray-bluish hue. Peripheral: Light-brown reticular area.
Crignis GSet al., 2013 [7]	77 years	Eczematous plaque with brownish-pink pigmentation, nipple retraction	Central: Whitish-pink area, irregular linear vessels, chrysalis-like structures. Peripheral: Dark brown pigmentation, blue-gray dots.
Hida T et al., 2014 [8]	62 years	Irregular black macules over right nipple	Dark-brown dots forming a reticular pigment network
Oliveira A et al., 2016 [9]	5 women (49–83 yrs)	4: eczema-like plaques; 1: fast-growing pigmented nodule	Pink-red background, polymorphous vessels, erosions, yellow scales, shiny-white streaks; chaotic brown dots, gray pigmentation
Apalla Z et al., 2019 [10]	62.4 ± 12.8 years (mean)	Retrospective study of 22 MPD lesions	Non-pigmented: Pink areas, white scales, dotted vessels, erosion, white shiny lines. Pigmented: Gray dots, pink areas, white lines.
Errichetti E et al., 2017 [11]	88 years	Serohematic crust on right nipple	Brownish-reddish crust, linear irregular vessels on pink-white background
Roš T et al., 2017 [12]	55 years	Erythematous, scaling plaque over NAC and adjacent skin	Central: Whitish-pink area with dotted/irregular vessels. Peripheral: Light brown pigmentation, pale streaks.
Shiomi T et al., 2018 [13]	66 years	Black macule (13 × 13 mm) with scaly surface	Central: Scaly surface, whitish-pink area. Peripheral: Irregular black dots, white halos.
Bhat Y et al., 2020 [14]	45 years	Erythematous-hyperpigmented indurated plaque over nipple	Central: Whitish-pink area with dotted vessels, chrysalis-like structures. Peripheral: White halos, fabric fibers, dark red areas.
Li C et al., 2020 [15]	67, 69 years	Dark red scaly patches with atrophic changes	Central: Brown scar, white-pink structure, bright stripes. Peripheral: Bright red background, white powdery areas, hairclip vessels.
Meena A et al., 2025 [16]	70 years	Bluish-black plaque with nipple destruction, reticular pigmentation, 2 satellite lesions	Grayish-black honeycomb-patterned pigment network, bluish-white veils, structureless pale-white areas, black pigmented dots
Oberoi B et al., 2025 [17]	75, 67, 54 years	Erythematous/brown proliferative NAC plaques, moist/crusted	Pink-orange background, yellow/white scales, dotted and linear vessels, blood spots, brown peppering, chrysalis structures
Robles-Tenorio A et al., 2024 [18]	69 years	Dark brown lesion with surrounding pink plaque	Central: Atypical pigment network, black granules/globules. Peripheral: Structureless pink/white areas.

These reports highlight a spectrum of dermoscopic patterns, including vascular, pigmentary, and structural alterations, which may aid in early recognition and differentiation of MPD from its clinical mimics. Notably, pigmented MPD often presents with features resembling melanoma - such as atypical pigment networks, regression structures, blue-gray dots, and polymorphous vessels - while non-pigmented cases typically show pink or white structureless areas, white scales, and chrysalis-like structures [3-9,14,16,18]. This variability underscores the heterogeneity in clinical presentation and dermoscopic appearance across cases. It also highlights that central areas of MPD often show pink-whitish structureless zones and chrysalis-like lines, while peripheral areas may exhibit diffuse pigmentation, linear vessels, and brown dots, especially in chronic or late-stage disease [6,7,12-15,18]. In our case, dermoscopy revealed white scales, a pink structureless background, and white shiny lines-findings consistent with non-pigmented MPD.

Differentiating MPD from its common mimickers using dermoscopy is clinically crucial. Eczema typically displays yellow-white crusts over a faint pink background, with dotted, comma-shaped, or patchy linear vessels, reflecting chronic inflammation. Psoriasis usually presents with white scales on a homogeneous pink background, accompanied by uniform dotted vessels, although it rarely involves the NAC. Bowen’s disease often features surface scales, clustered glomerular vessels, and hyperkeratosis but generally lacks pigmentation [17]. Recognizing these distinct dermoscopic features, as summarized in Table 2, is crucial in avoiding misdiagnosis in clinically ambiguous cases.

**Table 2:** Comparison of dermoscopic features of mammary Paget’s disease (MPD) and its mimickers

Feature	Mammary Paget's Disease	Eczema	Psoriasis	Bowen’s Disease
Vascular morphology	Irregular linear, dotted vessels, sometimes blood spots	Dotted vessels, patchy distribution	Dotted vessels, regular distribution	Glomerular, clustered, coiled, dotted vessels; linear at periphery
Scale characteristics	Fine white scales, yellow-white scales	White and yellow scales (often both), diffuse or focal	Predominantly white scales, diffuse	Scaly surface, can be thick
Background color	Pink structureless areas; erythematous moist plaque	Yellowish or dull red, sometimes light red	Light red or bright red background	Structureless hypopigmented or pink areas
Other structures	Shiny white lines/streaks (chrysalis), blue-grey dots/peppering	Brownish-orange dots, yellow-orange clods	Non-specific	Small brown globules; structureless grey or brown pigmentation
Distribution of structures	Focal or diffuse; parallel or orthogonal white lines	Patchy distribution of vessels and scales	Regular distribution	Asymmetric; pigmented structures often linear or clustered
Ulceration or erosion	Common (often with surface crusting)	Uncommon	Rare	Occasional, especially in advanced lesions
Pigmented variant	Diffuse irregular pigmentation, regression structures, pigmented granules	Not typical	Not typical	Pigmented variant: brown dots, homogeneous gray/brown

Histopathologically, MPD is characterized by large pale-staining cells with hyperchromatic nuclei - referred to as Paget cells - distributed within the epidermis [1,2]. Although Bowen’s disease can exhibit pagetoid spread and may appear similar histologically, the absence of full-thickness epidermal atypia and epidermal hyperplasia, along with a specific immunohistochemical profile, helps distinguish the two. In this case, the tumor cells were strongly positive for cytokeratin 7 (CK7) and HER2 and negative for cytokeratin 5/6, thereby confirming the diagnosis of MPD and ruling out Bowen’s disease.

Surgical intervention remains the mainstay of treatment for MPD. While mastectomy was traditionally the standard, breast-conserving surgery (BCS) involving nipple excision followed by radiotherapy is now preferred in patients without underlying carcinoma. Prognosis is significantly influenced by the presence or absence of invasive disease. Patients without a palpable mass have favorable outcomes, with five- and ten-year survival rates of 92% and 82%, respectively [19]. In contrast, those with palpable invasive carcinomas have reduced five- and ten-year survival rates of 38% and 22%, respectively [19].

For patients who are not surgical candidates or who decline surgery, non-invasive therapies are increasingly being considered. Topical imiquimod, an immune-response modifier, has shown promise in treating both MPD and extramammary Paget’s disease. It enhances local immune activity, promoting the destruction of malignant cells. In our case, the patient was treated with imiquimod 5% cream, applied three times per week for 16 weeks, resulting in moderate clinical improvement. Other emerging treatments include photodynamic therapy (PDT), which utilizes a light-sensitive agent and targeted light exposure to selectively destroy neoplastic cells, and topical 5-fluorouracil, although evidence supporting its use remains limited. Radiation therapy may also be an option for patients who cannot undergo surgery [1,2,19].

## Conclusions

This case reinforces the importance of considering MPD in patients with persistent, treatment-resistant nipple eczema. A clinico-dermoscopic-pathologic correlation (CDPC) approach remains vital for early and accurate diagnosis. Dermoscopy is not only helpful for diagnosis but also for monitoring therapeutic response and identifying recurrences. Future integration of advanced imaging tools such as reflectance confocal microscopy (RCM) and optical coherence tomography (OCT) may further improve diagnostic precision, especially in atypical or pigmented cases.
